# RAS pathway targeted therapy in patients with *DICER1*-associated sarcomas

**DOI:** 10.1038/s41698-025-01026-0

**Published:** 2025-07-09

**Authors:** Lindy Zhang, Paige H. R. Mallinger, Serena Zhou, Stavriani C. Makri, John M. Gross, Calixto-Hope G. Lucas, Ying S. Zou, William Mize, Damon R. Olson, Senna R. Munnikhuysen, Jawhar Rawwas, Yoav Messinger, Kenneth S. Chen, Kris Ann P. Schultz, Christine A. Pratilas

**Affiliations:** 1 Department of Oncology, Sidney Kimmel Comprehensive Cancer Center, Johns Hopkins University School of Medicine, Baltimore, MD USA; 2 Cancer and Blood Disorders, Children’s Minnesota, Minneapolis, MN USA; 3 International Pleuropulmonary Blastoma/DICER1 Registry, Children’s Minnesota, Minneapolis, MN USA; 4 Department of Pediatrics, University of Texas Southwestern Medical Center, Dallas, TX USA; 5 Department of Pathology, Johns Hopkins University School of Medicine, Baltimore, MD USA; 6 Department of Radiology, Children’s Minnesota, Minneapolis, MN USA; 7 Department of Pathology and Laboratory Medicine, Children’s Minnesota, Minneapolis, MN USA; 8 Department of Pediatrics, University of Kentucky, Lexington, KY USA

**Keywords:** Sarcoma, Targeted therapies, Paediatric cancer, Cancer genetics, Cancer genomics

## Abstract

*DICER1*-associated sarcomas commonly exhibit cooperating mutations involving RAS signaling pathways, but the efficacy of therapies that target these mutations is unknown. Here we report two children with *DICER1* tumor predisposition who presented with *DICER1*-associated sarcomas with cooperating, targetable mutations in *HRAS* or *BRAF*. Both had relapsed/progressed disease despite upfront multimodal therapy and were subsequently treated with molecularly targeted agents. In the first case, mutant *BRAF* became amplified after dual dabrafenib/trametinib therapy, presumably as a driver of acquired resistance. In the second case, a subclonal *HRAS* variant at diagnosis became the predominant clone at autopsy, suggesting its importance in therapy resistance. Together, these two cases provide molecular evidence of the significance of RAS/ERK signaling in *DICER1*-driven tumorigenesis and highlight the potential for targeting these cooperating mutations.

## Introduction

*DICER1* tumor predisposition (OMIM 606241) is caused by pathogenic variants (PVs) in the *DICER1* gene. Germline *DICER1* variants were first detected in individuals affected with familial pleuropulmonary blastoma (PPB), a sarcoma of the lungs in childhood^1^. Subsequently, the International PPB/*DICER1* Registry and others have reported additional conditions associated with *DICER1* including multinodular goiter of the thyroid, differentiated thyroid carcinoma, pituitary blastoma, pineoblastoma, ciliary body medulloepithelioma, nasal chondromesenchymal hamartoma, cystic nephroma, Wilms tumor, ovarian Sertoli-Leydig cell tumor (SLCT), and cervical embryonal rhabdomyosarcoma^2,3^. Recently, characteristic histopathology findings and distinct methylation signatures have been described in *DICER1*-associated sarcomas in variable anatomical locations and lend strong evidence that they constitute a distinct sarcoma entity^4–6^. *DICER1-a*ssociated sarcomas often demonstrate unique histopathologic features of variably cellular spindled cells accompanied by rhabdomyoblastic differentiation and occasional cartilaginous differentiation^4,7^.

The human *DICER1* locus is located on chromosome 14q32.13. DICER1 is a cytoplasmic endoribonuclease within the canonical microRNA (miRNA) biogenesis pathway and is critical for the proper processing of precursor microRNA (pre-miRNA) double-stranded hairpins to their mature single-stranded forms^8^. The protein utilizes its ribonuclease (RNase) IIIa and IIIb domains to process a pre-miRNA into mature miRNAs. These mature miRNAs are then loaded into an Argonaute protein to form an RNA-induced silencing complex (RISC) to ultimately downregulate an mRNA target transcript through base-pair complementarity. miRNAs regulate genes involved in a variety of biological processes, including stem cell maintenance, organogenesis, and oncogenesis; therefore, failed miRNA processing is a key tumorigenic event in *DICER1*-mutant tumors^9^. In most cases, germline PVs in *DICER1* are loss-of-function variants that can arise anywhere in the gene, while second-hit somatic mutations in tumors are nearly always limited to exons 24 and 25 that encode the RNase IIIb domain^9^. The RNase IIIa domain cleaves on the 3p side of pre-miRNA hairpins, and the RNase IIIb domain cleaves on the 5p side. For this reason, somatic mutations in the RNase IIIb domain lead to impaired production of “5p-derived” miRNAs.

It is established that the immediate molecular consequence of RNase IIIb loss-of-function mutations is an imbalance of 3p and 5p miRNAs; however, the downstream consequences that lead to tumorigenesis are unclear. Cooperative oncogenic alterations are often seen in *DICER1*-associated tumors. Biallelic inactivation of *TP53* is the most common co-occurring event seen in *DICER1*-mutant sarcomas, and genetic alterations in *NF1*, *KRAS*, *NRAS*, *FGFR4, EGFR*, and *PDGFRA* have also been reported^5,10–14^. These findings suggest that oncogenic RAS/extracellular signal-regulated kinase (ERK) signaling may have a role in the tumorigenesis of *DICER1-*associated sarcomas and, if so, could represent potential therapeutic targets. There is currently no universally accepted systemic therapy approach for the treatment of patients with *DICER1*-associated sarcomas. Instead, standard regimens used for other cancers, such as rhabdomyosarcoma, germ cell tumor, or soft tissue sarcoma, are most often the treatment paradigms chosen for specific *DICER1*-associated cancers. As additional knowledge is gained regarding the molecular characterization of *DICER1*-associated sarcomas, an evaluation of the use of molecularly targeted therapies as precision-driven therapeutic options is necessary.

Here, we report the clinical experience of two patients with confirmed recurrent and metastatic *DICER1*-related malignancies who received RAS pathway targeted therapies on the basis of identified cooperating mutations in their tumors. In these two patients, clinical genomic sequencing guided the use of precision-targeted therapy following standard sarcoma or solid tumor regimens. Our experience highlights the safety and potential application of RAS pathway inhibitors in patients with these tumors and suggests an opportunity to further explore the efficacy of these treatments in similar patients.

## Results

### Patient 1

**Fig. 1:**
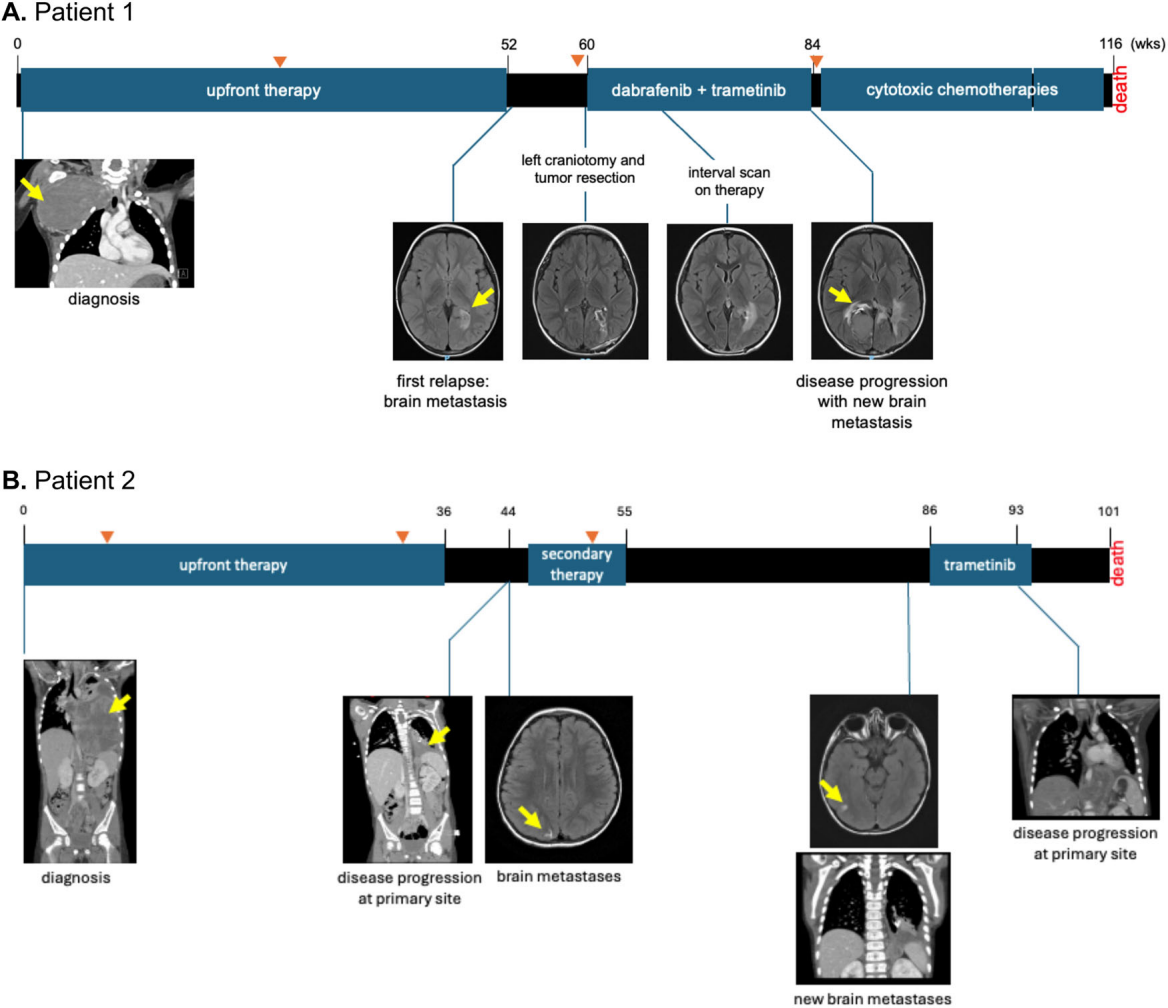
Timeline of the clinical course for each patient. **A** Patient 1, with *DICER1*-associated sarcoma and BRAF p.V600E; and **B** Patient 2, with pleuropulmonary blastoma and HRAS p.G13R. MRI imaging at key timepoints is shown, including available imaging during treatment with molecularly targeted agents (dabrafenib/ trametinib, Patient 1; trametinib, Patient 2) as indicated. Yellow arrows indicate tumor location. Orange arrows indicate surgical intervention.

A 3-year-old girl presented with a mass on her right upper chest extending into the shoulder. The mass grew rapidly over a period of approximately 6 weeks and caused progressive loss of right hand function and significant pain in the right arm. Past medical history and family history were unremarkable. A chest CT demonstrated a large right anterior superior chest mass with remodeling of the surrounding osseous structures and encasement of the right brachial plexus, including the right C6-T1 nerve roots and the subclavian artery (Fig. 1A). Multiple pulmonary nodules were identified, ranging from 3 to 5 millimeters (mm), along with two cysts (8 mm and 5 mm) at the right lung base. There was no other radiographic evidence of distant metastases. Open biopsy of the primary lesion revealed a high-grade primitive spindle cell sarcoma with pleomorphism; sequencing of the tumor specimen via the Johns Hopkins Molecular Diagnostic Laboratory in-house next-generation sequencing (NGS) detected two *DICER1* variants (p.E1813D and p.M1428fs) and a *BRAF* p.V600E mutation (“Primary” in Table 1). Germline testing in the patient confirmed the germline origin of the *DICER1* p.M1428Cfs variant, which was subsequently also identified in paternal germline testing. Her mother and newborn sister were negative for this *DICER1* variant.

**Table 1:** Identified genomic alterations in each specimen

Patient 1					Patient 2				
		Primary	First recurrence	Second recurrence			Primary	Primary subclone	Autopsy
		Variant allele frequency (%) in the specimen					Variant allele frequency (%) in specimen		
*DICER1*	p.E1813D	50.25	43.76	47.78	*DICER1*	p.G1809R	present	32.2	44.8
*DICER1*	p.M1428fs	45.54	44.84	42.71	*DICER1*	p.R676*	48.2	55.1	35.3
*BRAF*	p.V600E	47.98	42.86	90.78	*HRAS*	p.G13R	43	2	65.2
*ATM*	p.L950F	46.34	48.77	46.08	*TP53*	p.C242F	17	0^a^	0^a^
*TP53*	c.559+2 T > C	7.45	–		*TP53*	single-copy loss	detected		detected
*FLT1*	p.T256	28.21	33.04	30.19	*TP53*	c.993+1 G > T		40	0^b^
*LEF1*	p.V145L	47.69	46.69	48.25	*MED12*	p.Q2073fs	detected^c^		
*NOTCH1*	p.P284L	47.46	51.03	51.43	*CACNA1D*	p.Q2031H	detected^c^		
*NSD2*	p.P1343A	51.47	51.7	96.83	*EXT1*	p.E125*	detected^c^		
*PBRM1*	p.N333S	47.08	42.61	42.23					
*MKI67*	p.G282V			45.11					
*PCLO*	p.T2921I			30.08					
Other variants			*BRAF* amplification, *EZH2* equivocal amplification		Other variants	IGF1R amplification	(see Supplementary Table 1)	(see Supplementary Table 1)	
Tumor mutational burden (mutations/megabases)		2.64	*3.59*	3.53					

^a^*TP53* p.C242F variant was not detected at a sequencing depth of 61 or 21.

^b^*TP53* c.993+1 G > T, this region had a sequencing depth of 12 and was not adequately covered for the autopsy specimen.

^c^Additional alterations detected without reported allele frequencies on clinical NGS due to absence of known clinical actionability.

**Fig. 2:**
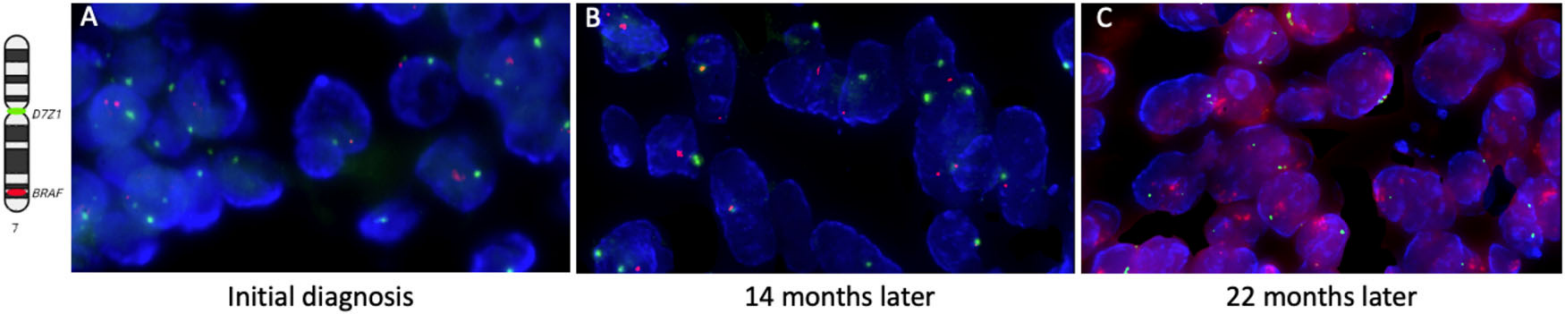
BRAF amplification was assessed using FFPE interphase FISH. Red signals represent the BRAF gene (7q34), and green signals correspond to the chromosome 7 centromere probe (D7Z1). **A**, **B** No BRAF amplification (copy-neutral status) is observed in interphase FISH of the initial diagnosis specimen (**A**) or a specimen collected 14 months later (**B**). **C** Interphase FISH demonstrates BRAF amplification, with more than ten red signals corresponding to the BRAF gene (7q34) per cell, alongside two green signals indicating two copies of the chromosome 7 centromere.

The patient was initially treated with multi-agent neoadjuvant and adjuvant chemotherapy following a high-risk rhabdomyosarcoma protocol (ARST0431^15^) with vincristine, irinotecan, doxorubicin, cyclophosphamide, ifosfamide, etoposide, and actinomycin-D, in addition to complete surgical resection and adjuvant radiation therapy to the primary tumor bed. Routine imaging at the completion of the planned 18-cycle treatment regimen revealed a new 1.3 by 1.2-cm lesion along the medial aspect of the left occipital horn of the lateral ventricle in the brain (Fig. 1A). A left craniotomy was performed to remove the tumor, which was confirmed again to be high-grade primitive spindle cell sarcoma. Johns Hopkins in-house NGS was performed and, again, detected the same *DICER1* variants and *BRAF* p.V600E mutation (“First recurrence” in Table 1), suggesting its origin as a recurrence of the chest mass rather than a new primary intracranial tumor. Post-operative brain MRI showed additional areas of leptomeningeal enhancement along the right parietal lobe, concerning for residual tumor. The patient received cranial radiation to both sites. She was treated with dabrafenib and trametinib for six months, until a recurrent intracranial tumor was detected on MRI imaging. Dabrafenib and trametinib were discontinued, and a maximally safe second surgical resection was performed. NGS findings were similar to the previous recurrence, with the same *DICER1* p.E1813D and BRAF p.V600E mutations, but in this specimen, amplification of the *BRAF* gene was also identified (Fig. 2 and “Second recurrence” in Table 1). Third-line chemotherapy was initiated with topotecan and temozolomide^16^ with additional proton radiation to the residual tumor. A third recurrence of intracranial disease was identified two months later. Given the 6-month progression-free interval associated with dabrafenib and trametinib, the *BRAF* p.V600E amplification was suspected to be a mechanism of resistance to type 1 RAF inhibition. To overcome this resistance, treatment with tovorafenib was pursued via single-patient compassionate access (prior to its Food and Drug Administration (FDA) approval). While waiting for approvals, she was treated with a regimen consisting of cyclophosphamide and vinorelbine, but she rapidly developed further clinical progression. She was enrolled in hospice care and died shortly after discontinuation of disease-directed therapy without having received additional RAF-targeted therapy.

### Patient 2

A 4-year-old girl presented with fever, abdominal pain, weight loss, and neck pain. A chest CT demonstrated a large left posterior chest mass with significant mass effect on the mediastinum (Fig. 1B). Metastatic disease was noted in the left kidney and adjacent lymph nodes, iliac bone, and thoracic and cervical spine, the latter extending into the neural foramen and encasing the vertebral artery. An open biopsy of the pulmonary lesion revealed Type II/III PPB. The patient was initially treated with multi-agent neoadjuvant and adjuvant chemotherapy including ifosfamide, vincristine, actinomycin D, and doxorubicin (IVADo^17^). She underwent surgical resection with intraoperative spillage and gross residual disease. Sequencing of the tumor specimen via GEM^TM^ Cancer Panel detected a *DICER1* variant (p.G1809R) and the *HRAS* p.G13R mutation (“Primary” in Table 1). Further germline testing confirmed the germline origin of the *DICER1* p.R676* variant.

Near the end of upfront therapy, surveillance imaging demonstrated a 2 cm structure in the medial inferior left chest along the diaphragm, along with persistent left adrenal and osseous metastases (Fig. 1B). A brain MRI revealed new focal enhancement in the right posterior parietal/occipital region. She received radiation therapy to the left chest and upper abdomen. Resection of the intracranial lesion confirmed metastatic PPB. She was then treated with vincristine, oral irinotecan, and temozolomide and radiation therapy to the right occipital lobe and subsequently developed two new metastatic lesions in the right temporo-occipital region and along the right calcarine fissure of the brain. She received additional radiation to these sites. Trametinib was added to her treatment in an attempt to target the *HRAS* mutation, and a response was noted at four weeks (Fig. 1B). However, further progression developed rapidly at 6 weeks following initiation of trametinib, and she died from intracranial hemorrhage and progressive thoracic disease 13 months after diagnosis of the first intracranial metastatic lesion.

Retrospective sequencing of lobectomy and postmortem tumor samples (posterior mediastinum and retroperitoneum) (“Primary subclone” and “Autopsy”, respectively, in Table 1 and Supplementary Table 1) showed *HRAS* p.G13R in both specimens, without any copy number changes at the *HRAS* locus. In the primary subclone, however, while we detected the oncogenic *DICER1* variant at a high variant allele frequency (VAF), we only detected *HRAS* p.G13R at a low variant allele frequency, suggesting that this variant was only present at a subclonal level in the initial specimen, indicating intra-tumoral heterogeneity.

## Discussion

Germline *DICER1* PVs increase an individual’s risk for benign and malignant tumors. Many of these are sarcomas, including PPB, embryonal rhabdomyosarcoma (ERMS), anaplastic sarcoma of the kidney, and primary intracranial sarcomas (PIS), among others^2,3^. There has not been a prospective clinical study to determine the optimal management of non-PPB *DICER1*-associated sarcomas, given the rarity and heterogeneity of these cancers; however, regimens used for PPB, such as VAIA (vincristine, actinomycin-D, ifosfamide, doxorubicin) and IVADo, are often used for *DICER1*-associated sarcomas. Here, we report on the clinical outcomes of two patients with metastatic *DICER1*-associated sarcomas who had transient responses to molecularly targeted therapies used in the relapsed setting. Our clinical experiences highlight the potential of targeted therapeutics in the management of *DICER1* sarcomas with known targetable mutations.

Retrospective studies have attempted to assess clinical outcomes associated with chemotherapy regimens in the treatment of *DICER1*-associated malignancies. The European Cooperative Study Group for Pediatric Rare Tumors (EXPeRT) analyzed a cohort of 52 pediatric patients with Type II or III PPB and reported that all patients received chemotherapy, including mostly standard rhabdomyosarcoma regimens^18^. Moreover, patients who received doxorubicin-containing regimens (IVADo or VAIA) had significantly better 5-year progression-free survival (PFS) compared to all other regimens (70% versus 31.3%, *p* = 0.01). In a retrospective analysis from the International PPB/*DICER1* Registry, outcomes associated with various chemotherapy regimens, specifically the IVADo regimen, were evaluated in 314 children diagnosed with Type II or III PPB who received upfront chemotherapy^17^. Compared to historical cohorts, treatment with IVADo was associated with an estimated 23% and 35% decrease in progressive disease and in death, respectively, although these differences were somewhat mitigated by trends in time. Despite intensive treatment, however, children with PPB type II and III with distant metastasis face 3-year overall survival (OS) of 40% and 0%, respectively^17^. Given the limited knowledge, many patients with *DICER1-*associated sarcomas are treated on similar general soft tissue sarcoma regimens^10,11^. A new prospective study for PPB is evaluating the impact of the addition of camptothecins to an IVADo-based regimen for newly diagnosed children with Types II and III PPB (Children’s Oncology Group (COG) ARAR2331).

A growing number of studies have unveiled recurrent genetic alterations in *DICER1*-associated sarcomas, which may suggest putative targets for therapeutic interventions. Mutations or deletions in *TP53* are the most common co-occurring genetic alterations found in *DICER1-*associated PPB and intracranial sarcomas^5,10,11,13^, but less commonly in SLCT or sarcomas within the genitourinary tract^11,12^. Genomic alterations in RAS signaling pathways are frequently found in *DICER1-*associated sarcomas. For example, *KRAS* and *NRAS* mutations have been identified in *DICER1*-mutated PIS, ERMS, and PPB^5,6,10,11,13^. Additional mutations identified in *DICER1*-associated sarcomas that lead to dysregulated RAS signaling pathways include inactivating alterations in *NF1*^5,10,11^ and *BRAF* (other than p.V600E)^11^. A recent comprehensive molecular study on *DICER1*-associated sarcomas identified recurrent alterations in *TP53* (32/80, 40%), *KRAS* (17/80, 21%), *NRAS* (6/80, 8%), and *NF1* (8/80, 10%)^6^.

Both patients in this series had *TP53* alterations in at least one tumor specimen as well as an oncogenic mutation in the RAS/ERK signaling pathway. Patient 1 had the *BRAF* p.V600E mutation, and Patient 2 had the *HRAS* p.G13R mutation. *BRAF* p.V600E represents the most frequent oncogenic mutation in BRAF and is found in about half of melanomas, 40% of papillary thyroid cancers, and about 10% of colorectal cancers^19^. While the majority of oncogenic *RAS* mutations are in *KRAS* (commonly found in pancreas, lung, and colon carcinomas), bladder urothelial carcinomas, head and neck squamous cell carcinomas, and rhabdomyosarcomas more frequently harbor *HRAS* mutations^20^. Oncogenic mutations in *HRAS*, *NRAS* and *KRAS* occur at similar hotspot codons, including G12, G13, and Q61, and less frequently A146^21^. Neither of the mutations identified in our patients (*BRAF* p.V600E or *HRAS* p.G13R) has been previously published in *DICER1*-associated malignancies, despite being bona fide driver mutations in other cancers.

It is not yet known how mutations in *DICER1* and in components of RAS pathways interact and perhaps amplify signals to drive tumorigenesis. Preclinical studies show that the addition of *Dicer1* mutations to *Ras*-mutated mouse models of malignancies confers accelerated tumorigenesis or decreased survival^22–24^. Furthermore, it has been reported that *Dicer1* haploinsufficiency promotes the development of distant metastases in a preclinical mouse model of undifferentiated pleomorphic sarcoma (UPS) expressing *Kras-G12D* but not those expressing *Braf-V600E*^23^, suggesting additional complexity in the way distinctive alterations within the RAS/ERK pathways cooperate with *DICER1* mutations. Alternatively, *DICER1* mutations in the RNase IIIb domain trigger increases in RAS/ERK signaling output genes in *DICER1*-mutated differentiated thyroid cancers, suggesting that dysfunctional *DICER1* alone drives RAS signaling pathways^25^. It is therefore reasonable to postulate that additional mutations within the RAS pathways potentiate dysregulated signaling and could lead to more aggressive behavior. Additional preclinical work suggests that RAS/ERK pathways also lead to phosphorylation, and thus activation, of DICER1, contributing to tumor development and invasion^26^. Additional studies are needed to gain deeper insight into the relationship between *DICER1* and specific RAS/ERK pathway mutations in malignancies.

The finding of genomic alterations in RAS/ERK pathways raises the potential for therapeutic intervention with targeted inhibition of its dysregulated signaling. A number of RAF inhibitors (RAFi) and MEK inhibitors (MEKi) are currently FDA approved for use in genomically selected cancers as well as Neurofibromatosis type 1 (NF1)-associated plexiform neurofibroma^19,27–29^. Patient 1 received the combination of dabrafenib (RAFi) and trametinib (MEKi), and Patient 2 received trametinib (MEKi), treatment recommendations that were guided by tumor genetic profiling (*BRAF* p.V600E and *HRAS* p.G13R, respectively). Both patients experienced relatively short periods of stabilized disease following targeted agent administration prior to the rapid progression of their disease burden. Previously published cases on the use of targeted agents in *DICER1*-associated malignancies include a young adult patient with an anaplastic sarcoma of the kidney with biallelic somatic *DICER1* mutations and concurrent activating *PDGFRA* p.D842V mutation, treated with avapritinib, resulting in significant response but intolerable toxicities^14^. An additional report highlighted a 2-year-old child with a histologic diagnosis of initially localized PPB (no germline or somatic *DICER1* variant was identified) who then had a second disease recurrence as brain metastases that demonstrated ETV6::NTRK3 fusion and was treated with larotrectinib for four cycles prior to progression of the disease and death^30^. In our cases, both patients had already received multimodal therapies and had aggressive clinical courses before targeted agents were used as treatment regimens. Therefore, we are unable to draw broadly applicable conclusions on the optimal timing and clinical scenario in which targeted therapeutics may be beneficial in these cancers. Additionally, it is not yet known if there is benefit to the use of molecularly targeted agents in combination with specific conventional chemotherapy or other agents such as immunotherapy, which has shown success in other cancer types^31–33^. Further studies are needed to understand the potential role of targeted therapies in this patient population, as there is not currently a uniformly accepted standard of care systemic treatment for patients with *DICER1*-associated sarcomas.

In these two cases, we further evaluated subsequent tumor specimens following treatment with targeted therapy, which allowed us to characterize molecular responses to the targeted therapy. In the post-treatment tumor specimen of Patient 1, there was emergence of *BRAF* copy number amplification, which is a known mechanism of resistance to type 1 RAF inhibition in cancer cells harboring the *BRAF* p.V600E mutation^34^. Based on these findings, we gained compassionate use access to tovorafenib, a pan-RAF inhibitor shown to overcome resistance to type 1 RAF inhibitors (e.g., vemurafenib, dabrafenib)^35^, but unfortunately, rapid intracranial disease progression precluded administration of this agent prior to her abrupt death. Conversely, in the post-treatment tumor specimen from Patient 2, the *HRAS* mutation remained detectable with a VAF of 65.2%. While MEK inhibition was used in both cases based on tumor genomic status, it is possible that a MEKi alone was insufficient to overcome the aggressive biology and advanced disease burden.

There is a growing repertoire of molecularly targeted agents directed at oncogenic RAS/ERK signaling, many in early phase clinical trials or approved in other RAS-driven cancer types. The clinical experiences described above are the first attempts to molecularly characterize clinical responses in patients with *DICER1*-associated sarcomas harboring RAS/ERK signaling pathway mutations. More preclinical and clinical studies are needed to determine the most effective, precision-driven therapeutic strategies for these rare cancers.

## Methods

### Case reports

Two pediatric patients treated for *DICER1*-associated sarcomas were identified for this case series. Retrospective chart review was conducted to extract clinical data relevant to each case. The research has been conducted in compliance with all relevant regulations including the Declaration of Helsinki. Written informed consent was obtained from the patients’ parents prior to publication of this case series.

### Next-generation sequencing (NGS)

NGS for Patient 1 was conducted by the Clinical Laboratory Improvement Amendments (CLIA)-certified Johns Hopkins Molecular Diagnostic Laboratory at the Johns Hopkins Hospital (Baltimore, MD), as described previously^36^. Targeted NGS was performed on formalin-fixed, paraffin-embedded (FFPE) malignant tissue sections to analyze the coding regions of cancer-related genes with the Johns Hopkins Solid Tumor Panel (https://pathology.jhu.edu/test-directory/ngs-solid-tumor-panel).

The primary tumor for Patient 2 had NGS conducted by the CLIA-certified clinical laboratory Ashion Analytics, LLC. Genomic DNA was extracted from the FFPE tumor sample and prepared using the KAPA Hyper Prep Kit (KAPA Biosystems) to create genomic libraries. Once the genomic libraries are created, a custom SureSelect XT Target Enrichment System (Agilent Technologies) is used to select specific genomic regions that are proprietary to the Ashion GEM Cancer Panel. Captured libraries are then clustered on a flow cell and sequenced using the Illumina HiSeq 2500 instrument. Sequence data were then analyzed using validated bioinformatic tools (Ashion pipeline version 2.0).

### Whole-exome sequencing

For the primary subclone and autopsy specimens from Patient 2, DNA was extracted from banked formalin-fixed, paraffin-embedded (FFPE) scrolls using the QIAamp DNA FFPE Tissue Kit (Qiagen, cat. 56404). Whole-exome sequencing and low-passage whole-genome sequencing were performed at Azenta Life Science (South Plainfield, NJ). Reads were aligned to the GRCh38 reference genome with BWA-MEM. Variants were called from whole-exome sequencing using Genome Analysis ToolKit (GATK) v3.7 and annotated using SnpSift. Copy number changes were called from low-passage whole-genome sequencing using CNVkit.

### Fluorescence in situ hybridization (FISH) on FFPE tumor specimens

FISH analysis was performed on interphase nuclei using a disease-specific probe panel targeting the *BRAF* gene, along with a centromere probe for chromosome 7 (D7Z1), following the FFPE protocol provided by the manufacturer (Empire Genomics Inc., Depew, NY, USA). A total of 100 nuclei were evaluated within the tumor areas marked by H&E staining. The assessment was performed by two independent technologists, who were blinded to each other’s results, using a Zeiss Axioscope fluorescence microscope (Zeiss Inc., White Plains, NY, USA). Data analysis was carried out using Cytovision software (Leica Inc., Buffalo Grove, IL, USA). The specimen was deemed abnormal if the observed results exceeded the laboratory-established threshold for the probe set.

## Supplementary information

Supplementary Table 1

## Data Availability

All data generated or analyzed during this study are included in this published article and its supplementary information files. Raw data files are available upon request from the corresponding author. NGS and WES data were generated will be deposited in dbGaP for public use.
