# Supplementary material for: RAS pathway targeted therapy in patients with DICER1-associated sarcomas
Source: NPJ Precis Oncol. 2025 Jul 9;9:232. doi: 10.1038/s41698-025-01026-0 (PMC12241403; doi:10.1038/s41698-025-01026-0)
Supplement: Supplementary file 1 — Supplementary Table 1 [file 41698_2025_1026_MOESM1_ESM.docx]

**Supplementary Table 1. Gene variants detected in primary subclone and autopsy specimen of Patient 2 via whole exome sequencing**

| **Gene** | **Variant** | **Primary Subclone** | **Autopsy** |
| --- | --- | --- | --- |
| ACMSD | p.Arg59Pro | + | - |
| ALDH1B1 | p.Arg107Ser | + | + |
| ATXN1 | p.His209Gln | + | + |
| BTNL3 | p.Ser397Arg | + | + |
| C9 | p.Gly83Arg | + | + |
| CASZ1 | p.Asp1703del | - | + |
| CCDC150 | p.Arg641His | + | + |
| CCDC157 | p.Arg31His | + | - |
| COL18A1 | p.Asp1279Asn | + | + |
| CPS1 | p.Thr350Pro | + | + |
| CYP3A7 | p.Trp408Ter | + | + |
| DICER1 | p.Arg676Ter | + | + |
| DICER1 | p.Gly1809Trp | + | + |
| DSC3 | p.Arg65Gln | + | + |
| DSPP | p.Asn1122Asp | + | - |
| DSPP | p.Asn1140_Ser1142del | + | - |
| EIF5B | p.Glu496del | + | + |
| ENGASE | p.Pro589Leu | - | + |
| ERBB4 | p.X179_splice | + | - |
| EXOC4 | p.Arg211His | + | - |
| FAM21A | p.Ala12Val | + | + |
| FCGBP | p.Asn3926Thr | + | - |
| FCGBP | p.Gln2735Pro | + | - |
| FMO2 | p.Ser195Leu | + | + |
| FRG2C | p.Cys12Arg | + | + |
| GNRH2 | p.Ser116ArgfsTer? | + | + |
| GPRIN2 | p.Arg5Cys | + | + |
| GPRIN2 | p.Ser104Ter | + | - |
| HRAS | p.Gly13Arg | - | + |
| HRCT1 | p.Leu100His | - | + |
| HSPA1L | p.Arg324Gln | + | + |
| IGSF3 | p.Asp1040_Asp1041insGlu | - | + |
| ISCU | p.Phe7Val | + | + |
| ITGB2 | p.Arg586Trp | + | + |
| KCNN3 | p.Gln78_Pro81dup | + | - |
| KRT32 | p.Asn402Ser | + | + |
| KRT84 | p.Arg184Leu | + | - |
| LAMC3 | p.Pro174Gln | + | + |
| LARS | p.Lys82Thr | + | + |
| LILRB3 | p.Arg466del | + | + |
| LPHN3 | p.Thr783Lys | + | + |
| MAGEA3 | p.Gly141Arg | + | + |
| MAGEB16 | p.Met248Glu | + | + |
| MAN2B2 | p.Arg374Gln | + | + |
| MFF | p.Ser7Phe | + | + |
| MRGPRX3 | p.Ser83Leu | + | + |
| MUM1 | p.Arg386Cys | + | + |
| NBPF20 | p.Cys42Ser | + | + |
| NIM1K | p.Arg77Gln | + | - |
| NOSTRIN | p.Gly530Glu | - | + |
| NOTCH2 | p.Pro2359Ser | + | + |
| NPSR1 | p.Glu307Lys | + | + |
| OBSCN | p.Ala1000Thr | + | + |
| OR10G2 | p.Arg187Pro | + | + |
| OR8S1 | p.Leu82Pro | + | - |
| PCDHB4 | p.Pro255Phe | + | + |
| PHF3 | p.Ala602Thr | + | + |
| PIWIL4 | p.Gln327Leu | + | + |
| PNMA3 | p.Gln426Arg | + | - |
| PSG3 | p.Leu30Pro | + | + |
| RAP1GAP2 | p.Ala346Thr | - | + |
| RIMS2 | p.Arg683Gln | + | + |
| SASH1 | p.Arg1171Leu | + | + |
| SGK223 | p.Pro1170_Ala1171dup | + | + |
| SGK223 | p.Ser349_Gly350dup | + | + |
| SIRPA | p.Val132Thr | + | + |
| SLC22A12 | p.Val388Met | + | + |
| SMARCA2 | p.Gln236_Gln238del | + | + |
| SPANXN3 | p.Asn24Lys | - | + |
| TANC1 | p.Thr1573Ala | + | + |
| TAS2R30 | p.Ile267Val | + | + |
| TBP | p.Gln95dup | + | - |
| TCF15 | p.Val114Leu | + | + |
| TMED3 | p.Arg91Gln | + | + |
| TMEM150A | p.Arg178Ter | + | + |
| TP53 | p.X307_splice | + | - |
| TPTE | p.Arg220Gly | + | - |
| TSHZ3 | p.Val831Ile | + | - |
| TTC30B | p.Arg448Leu | + | + |
| UBXN11 | p.Pro489_Gly514del | + | + |
| UGT2B7 | p.Tyr268His | + | + |
| UNC79 | p.Glu1386Ter | + | + |
| ZKSCAN3 | p.Lys200Ala | + | + |
| ZNF91 | p.Leu1164Ala | + | - |
